# Thioflavin T Lasing Probe for Mucin Detection in Simulated Tears as a Targeting Strategy for Brain Tumors

**DOI:** 10.1021/acschemneuro.5c00274

**Published:** 2025-05-15

**Authors:** Ewelina Jalonicka, Konstantin Rusakov, Grzegorz Szwachta, Piotr Hanczyc

**Affiliations:** † Institute of Experimental Physics, Faculty of Physics, 49605 University of Warsaw, Pasteur 5, 02-093 Warsaw, Poland; ‡ Faculty of Construction and Environmental Engineering, 49561 Warsaw University of Life Sciences, 02-776, Warsaw, Poland; § Center of Cellular Immunotherapies, 49561 Warsaw University of Life Sciences, 02-786 Warsaw, Poland

**Keywords:** brain tumor, mucin, tears, thioflavin T, lasing, Fabry-Pérot cavity

## Abstract

A novel optical approach for a noninvasive detection of mucins in tear fluid is proposed, aiming at the early diagnosis of brain tumors such as glioblastoma. Utilizing Thioflavin T (ThT) as a fluorescent probe, our study demonstrates that ThT selectively binds to mucins (modeled by MUC3) in DEMI water, artificial tears, and simulated tears. Steady-state and time-resolved fluorescence spectroscopy reveal that mucin binding induces a significant enhancement in ThT fluorescence and prolonged emission lifetime, indicative of restricted intramolecular rotation. Importantly, the application of Fabry-Pérot cavity lasing spectroscopy enabled the resolution of distinct spectral signatures of the ThT–mucin complex, including the emergence of dual lasing peaks and an increased lasing threshold in mucin-rich samples compared to controls. These optical fingerprints provide compelling evidence of specific ThT–mucin interactions that are not discernible with conventional fluorescence techniques. Our findings highlight the potential of the ThT probe and lasing method as a sensitive, noninvasive platform for detecting mucins in tears, offering a promising strategy for the early detection of glioblastoma.

## Graphical abstract

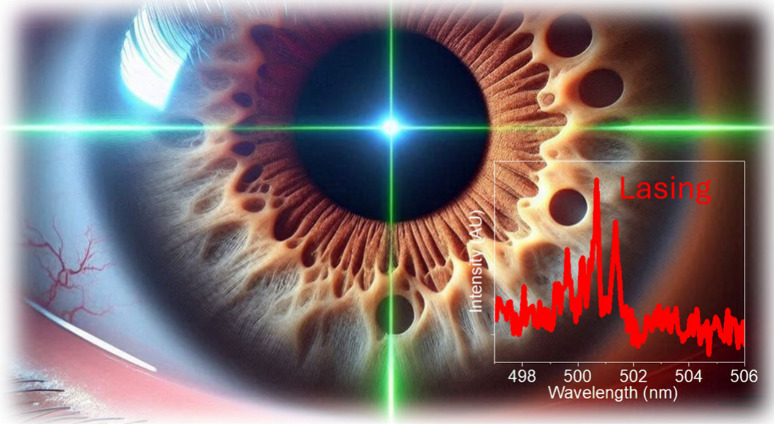

Mucins are large, highly glycosylated glycoproteins that are products of MUC genes and exhibit complex multidomain structures. Mucins are characterized by a high molecular weight (200 kDa – 200 MDa) and large size (Rg 10 – 300 nm), as well as a high degree of glycosylation (up to 90%).^1^ They are part of one of the three layers of the human tear film, the mucin layer, which is in turn surrounded by the aqueous and lipid layers. They are responsible for maintaining the stability of the tear film.^2^ There are two subtypes of mucins: secretory mucins (SM) and membrane-associated mucins (MAM) with different physicochemical properties.^2^ One of the fundamental characteristics of mucins is their capacity to form gels.^3^ This property is of significant physiological importance, as it enables the creation of chemical barriers that are important, for example, for the human immune system.^4,5^ Mucins play a relevant role in the pathogenesis of various pathological conditions, including infections,^6^ lung disease,^7^ and dermatological conditions.^8^ One of the most intriguing areas of research in mucins is their role in the pathophysiology of brain tumors, as evidenced by recent studies. Wang et al.^9^ observed an abnormally high expression of mucin 21 (MUC 21) in human tissues and cellular lines of glioblastoma, which was associated with tumor recurrence. A review of the literature reveals numerous associations between other types of mucins and glioblastoma. These include, for example, MUC 1, the inhibition of which has been demonstrated to induce cell cycle arrest and telomerase suppression in glioblastoma cells,^10,11^ as well as MUC 4,^12^ MUC 5AC, MUC 7,^13^ MUC 13,^14^ and MUC 16.^15^ It is important to note that brain and central nervous system (CNS) tumors are a diverse and extremely deadly group of malignant cancers. According to Global Cancer Statistics, there will be 308,102 new cases and 251,329 deaths from brain and CNS cancers in 2020.^16^ Among them, one of the most common and malignant diseases is glioblastoma (IV grade). It is a disease characterized by extremely poor prognosis—the 5-year survival rate is only 4–5%.^17^ Since mucins are not well structurally characterized, thus, we propose commercially available MUC 3 as a model system in a general concept of studying mucin-dye interactions for diagnostic purposes.

The dye used to stain MUC 3 was Thioflavin T (ThT), a well-known molecular rotor used in biomedical research. ThT has a high affinity to certain biomolecules and susceptibility to change in microenvironmet.^18–20^ ThT’s fluorescence properties are highly dependent on the rotational freedom of benzylamine and benzathiole rings.^21^ When ThT binds to biomolecules, the rotational motion of these rings is restricted,^22,23^ significantly enhancing fluorescence.^24^ This unique characteristic has made ThT a gold standard in the study of protein aggregation, particularly in research related to neurodegenerative diseases.^25^ It has been widely used to probe the transition of proteins from native to aggregated states, as it exhibits a marked increase in fluorescence fibrils.^26^ Recently, ThT’s ability to act as a reporter of the biomolecules microenvironment using lasing spectroscopy has sparked interest in its potential application beyond neurodegeneration.^27^ Like mucins, which report on the tumor microenvironment by modulating cellular signaling and barrier functions, ThT’s sensitivity to structural changes in its surrounding milieu positions it as a promising tool for studying mucin dynamics. Given that mucins are often overexpressed in cancers such as gliomas and metastatic brain tumors, leading to enhanced tumor growth, invasion, and immune evasion, ThT complexed with mucins could provide valuable insights into the biophysical changes that accompany mucin overexpression.^28^

In this study, we investigate the complexation of ThT with mucins dissolved in DEMI water, artificial tears, and simulated tears, utilizing a biophysical toolkit that includes lasing spectroscopy in Fabry-Pérot cavities^29^ to assess the sensitivity and selectivity of ThT to model MUC 3 in complex milieu. This approach aims to facilitate the *ex vivo* noninvasive detection of cancerous changes in patients who can potentially develop brain tumors.

Figure 1(a) presents the absorption and steady-state fluorescence spectra of the ThT-MUC 3 aqueous solution. Mucins exhibit a characteristic absorption band at 256 nm, while ThT displays a maximum absorption at 412 nm. Upon excitation at 405, 415, and 440 nm, a significant increase in emission intensity is observed, with a tendency for higher intensity at longer wavelengths (Figure 1(b)). These findings suggest that ThT exists in different conformations when interacting with mucins associated with a variety of binding sites for small molecules like ThT ((Figure 1(c)). One of them is electrostatic interaction of the positively charged ThT with negatively charged carboxylate groups (COO^–^) on the mucin molecule - similar mechanism was proposed for ThT binding to amyloid fibrils as one of the potential binding modes.^30^ Another binding mode reminiscent to amyloid fibrils is the hydrophobic character of β-sheet grooves whereby ThT is typically accommodated. Similar hydrophobic pockets are present in mucins (Figure 1(c)). In addition, hydrogen bonding can further stabilize ThT-mucin interaction, while the dye may also associate with oligosaccharide moieties and interact with cysteine-rich regions - potentially through the formation of disulfide bridges. Together, these multiple interaction modes present a variety of binding sites of ThT-MUC 3. Thus, ThT emissive species can be sensitive to probe structural changes in tears where mucins are indicators of potential cancer development.

**Figure 1:**
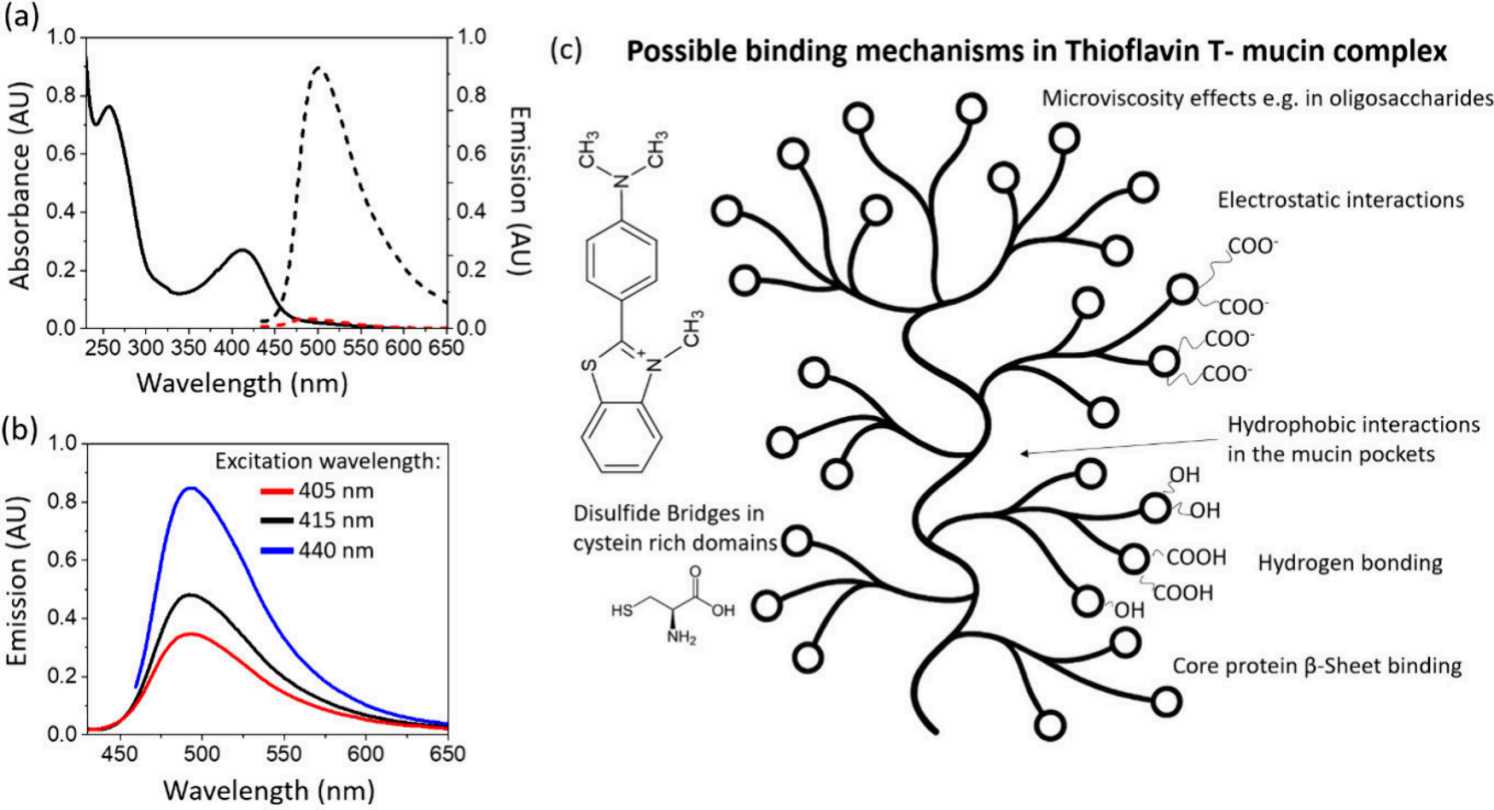
(a) Absorption (black solid) and fluorescence emission spectra of pristine Thioflavin T (ThT) in DEMI water (red dashed) and ThT in mixture with MUC 3 in DEMI water (black dashed), (b) emission of ThT with MUC 3 collected at different excitation wavelengths λ_ex_ = 405 nm (red), λ_ex_ = 415 nm (black), λ_ex_ = 440 nm (blue), (c) Structure of Thioflavin T (ThT) and illustration of mucin with possible binding mechanisms between ThT and mucin.

The ThT-mucin (MUC 3) solution was suspended in the artificial tears solution containing trehalose, sodium hyaluronate, sodium chloride, trometamol, hydrochloric acid, and binding of ThT to mucins was verified using time-resolved fluorescence spectroscopy. This method is particularly advantageous for assessing binding of whether the ring rotation of ThT is inhibited due to steric hindrance like it is in β-sheet groove binding of amyloid fibrils.^31^

Biexponential fitting of the fluorescence decay data revealed that ThT in the presence of mucins was significantly prolonged in comparison with the sample without MUC 3 (Figure 2). The addition of MUC 3 into the ThT-artificial tears solution suppresses the rotation of ThT aromatic rings, leading to extension of lifetimes to values of 0.39 ns (45%) and 1.74 ns (55%).

**Figure 2:**
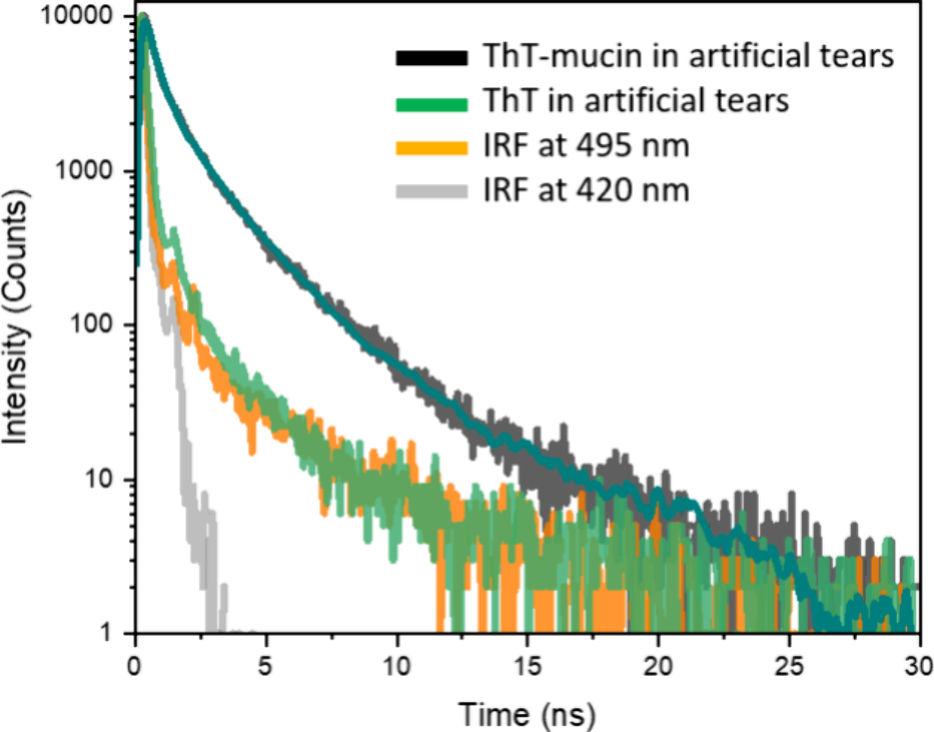
Fluorescence decay of ThT with mucin in artificial tears (black decay with cyan fitting curve). For comparison, the decay of ThT dissolved in tears (green) and the instrument response function (IRF, at 420 nm (gray) and 495 nm (orange)), Samples were excited λ_ex_ = 420 nm, and lifetimes were collected at λ_em_ = 495 nm.

Next, the ThT - mucin (MUC 3) solution was dispersed in a simulated tear solution. Unlike earlier versions of artificial tears, this formulation contains common components found in human tears, such as proteins: lysozyme, gamma-globulin, and bovine serum albumin. Each aforementioned component may interact with ThT, providing a competitive binding mechanism to MUC 3. Additionally, the presence of d-glucose and collagen increases the microviscosity of the solution in comparison to artificial tears, a parameter to which ThT is particularly sensitive.^32^ (*details on simulated tears composition see* SI: materials, and methods *)*.

Steady-state and time-resolved fluorescence spectroscopy provide useful insights into ThT interactions with single biomolecule, but they are not selective for multicomponent solutions, such as simulated tears. Lasing emission spectroscopy offers sensitivity and selectivity that enables discrimination of ThT bound to MUC 3 in the simulated tear solution (Figure 3). Lasing in Fabry- Pérot cavity can occur only in the condensed phase of biomolecules and dyes when there is sufficient optical gain. Two major requirements for lasing to occur are that gain must exceed losses, and there must be efficient feedback from the cavity mirrors.^33^ In the condensed phase of biomolecules and dyes, the high density of emitters, improved optical feedback, reduced nonradiative decay, optimal optical confinement, and favorable molecular conformations work collectively to satisfy these conditions. In less dense or diluted solution, these factors are not sufficiently pronounced, which prevents the establishment of the necessary population inversion and coherent optical amplification required for lasing.

**Figure 3:**
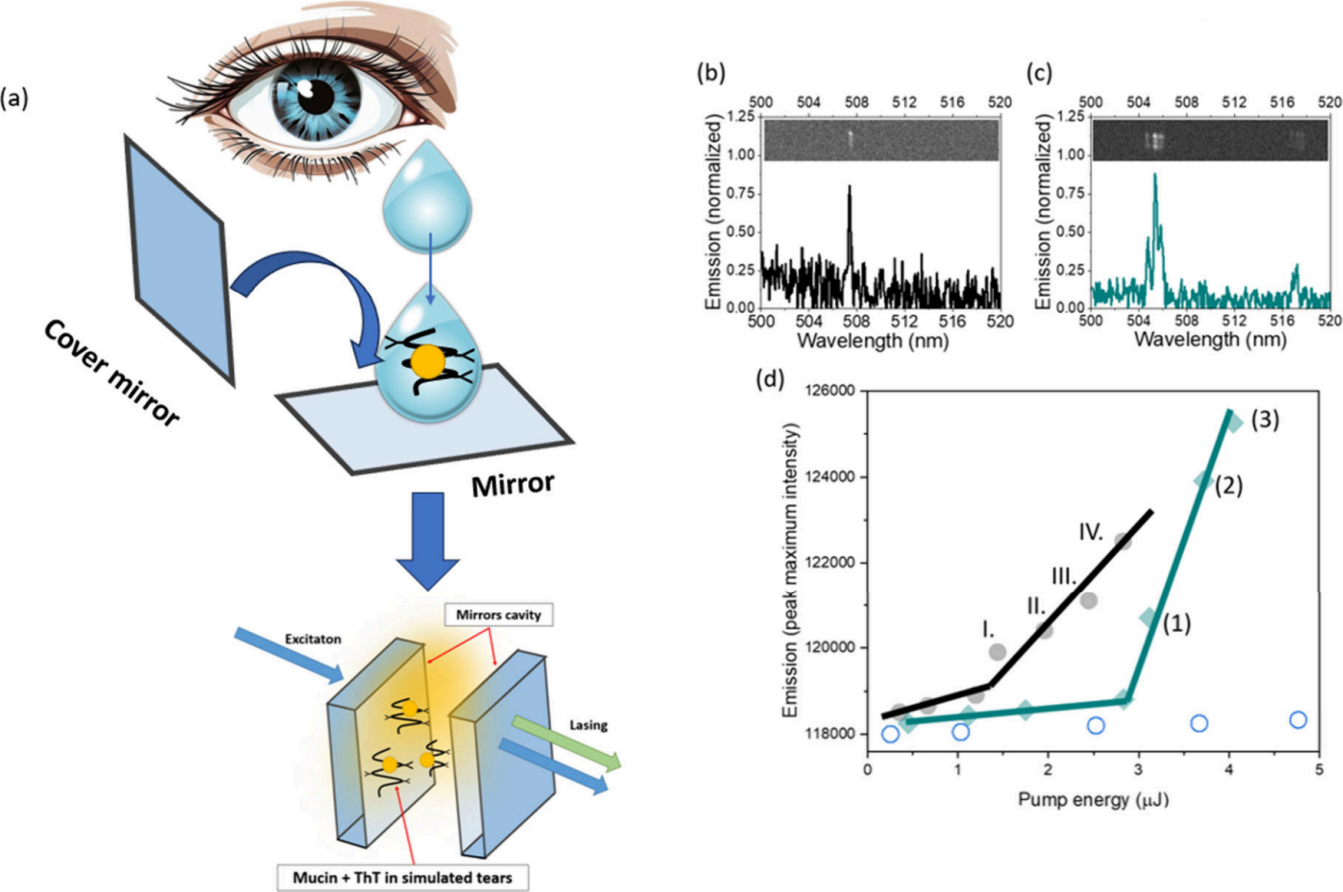
(a) Schematic representation of the sample preparation, where the Thioflavin T-mucin complex is suspended in simulated tears and the mixture is drop-cast onto one mirror, followed by the placement of a second mirror to form a Fabry-Pérot cavity. The solution serves as the gain medium for the lasing effect, (b) lasing spectrum of ThT in simulated tears solution, and (c) lasing emission spectrum of ThT with additions of mucins (0.2 mg/mL) to simulated tears solution. The top panels in lasing spectra are the output measurements, showing bright spots above the lasing threshold. (d) Plot of pump energy versus emitted intensity, illustrating the exponential rise in intensity once the lasing threshold is surpassed. Black dots are ThT dissolved in simulated tears and cyan diamonds are ThT-mucin in simulated tears. Open circles represent a control lasing experiment of ThT in simulated tears without condensation where no lasing was detected. Lasing was measured in a condensed phase done by the 10-fold reduction of volume using column filters. The numbering in (d) refers to the spectra shown in Figure S1 in Supporting Information: lasing in Fabry-Pérot Cavities. λ_ex_ = 420 nm, C_ThT_ = 25 mg/mL.

The sample for lasing was prepared by first concentrating simulated tears and tears supplemented with 0.2 mg/mL MUC 3 (matching human tear mucin levels which, based on the literature found, are estimated at approximately 0.2 mg/mL^34–36^ ), using 3 kDa column filters and centrifugation, which reduces the volume 10-fold. This concentration step enhances the interactions between ThT and the biomolecules. The concentrated solution was then drop-cast onto one mirror and covered with a second mirror to form a closed and planar cavity, where multiple reflections enable population inversion in high density of ThT emitters interacting with biomolecules (Figure 3(a)).

Unlike steady-state and time-resolved fluorescence techniques, which gather broadband emission signal, the lasing approach reveals narrowband emission peaks originating from ThT with restricted molecular rotation. Figure 3(b) illustrates the lasing emission spectrum of ThT in a simulated tear solution without MUC 3, whereas Figure 3(c) presents the spectrum obtained with the addition of MUC 3. In the presence of MUC 3, the lasing emission spectrum of ThT in the simulated tear mixture exhibits two distinct peaks (approximately 505 and 517 nm), whereas only a single peak (∼507 nm) is observed in the absence of MUC3. The difference in lasing thresholds has been also found. ThT-MUC 3 solution requires higher pump energy (threshold ∼ 3.0 μJ) to generate lasing action compared to ThT in MUC 3-free tears (threshold ∼ 1.4 μJ) (Figure 3d). Together, this two-parameter lasing response, manifested as both a shift in lasing wavelength and an increase in lasing threshold, highlights subtle changes in ThT photophysical behavior induced by MUC 3 binding. The interaction of MUC 3 with dye is much more complex than ThT binding with amyloid fibrils, where ThT selectively binds to β-sheet grooves. These include interactions with the MUC 3 core protein, oligosaccharide side chains, and specific cysteine-rich domains. Thus, lasing spectroscopy of ThT-MUC 3 solutions brings new information about complex binding mechanisms, providing compelling evidence of sensitive and selective distinction of MUC 3 in complex tears milieu indiscernible by conventional ensemble fluorescence techniques.

In summary, the mechanism of the ThT-mucin bindings has not been studied and is therefore unknown. Our studies show the potential for practical use of ThT-mucin research in medicine. It is demonstrated that the complexation of ThT with MUC 3 leads to significant fluorescence enhancement and prolonged fluorescence lifetimes. ThT was examined in various aqueous environments, including DEMI water, artificial tears, and simulated tears. The findings indicate that ThT-MUC 3 interactions mimic the dye’s behavior in other rigid binding environments (such as amyloid fibrils)^37^ but with multiple binding mechanisms due to MUC 3 structural diversity. Lasing spectroscopy brings spectral signatures of ThT-MUC 3 binding in the complex milieu of simulated tears. Such changes in the lasing readouts i.e. emergence of distinct emission lines alongside variations of lasing threshold, serve as unambiguous optical fingerprints of MUC 3 detection highlighting the potential of lasing for identifying mucins in tears with high specificity.

The present study introduces a promising strategy for noninvasive diagnostics of brain tumors by targeting tear fluid. The ability to detect MUC 3 in tear-like fluids suggests that tear samples from patients could be screened for aberrant mucin signatures associated with glioblastoma. Such a diagnostic approach would be minimally invasive, relying on simple tear collection (e.g., Schirmer strips^38^) methods and optical analysis of a ThT-doped tear sample. Looking forward, integrating this lasing-based probe with established tear sampling techniques could become a simple and robust screening method for clinically relevant mucins. By combining its molecular specificity, optical amplification, and noninvasive sampling, this concept opens the avenues for early detection of glioblastoma and potentially other conditions where mucin dynamics reflect disease state.

## Supplementary Material



## Notes

Materials, preparation protocols for fluorescence and lasing experiments, methods descriptions and Figure S1 Lasing spectra of ThT in simulated tears as a function of pump energy (PDF)
