# Supplementary material for: Thioflavin T Lasing Probe for Mucin Detection in Simulated Tears as a Targeting Strategy for Brain Tumors
Source: ACS Chem Neurosci. 2025 May 15;16(13):2371–5. doi: 10.1021/acschemneuro.5c00274 (PMC12232303; doi:10.1021/acschemneuro.5c00274)
Supplement: Supplementary file 1 [file cn5c00274_si_001.pdf]

Thioflavin T Lasing Probe for Mucin Detection in Simulated Tears as a Targeting Strategy for Brain Tumors

Ewelina Jalonicka[1], Konstantin Rusakov[2], Piotr Hanczyc[1,3]

[1] Institute of Experimental Physics, Faculty of Physics, University of Warsaw, Pasteura 5, 02-093 Warsaw, Poland

[2] Faculty of Construction and Environmental Engineering, Warsaw University of Life Sciences, 02-776, Warsaw, Poland

[3] Center of Cellular Immunotherapies, Warsaw University of Life Sciences, 02-786 Warsaw, Poland

*Correspondence to: piotr_hanczyc@sggw.edu.pl

Materials:

Thioflavin T: was "UltraPure Grade" purchased from AnaSpec (USA). Two stock solutions were prepared in DMSO: for steady-state and time-resolved fluorescence the stock solution was of 1 mg/mL whereas for lasing experiments was 25 mg/mL.

Mucin 3 (MUC 3): was purchased from Sigma Aldrich. It is mucin type III, bound sialic acid 0.5 – 1.5% (from porcine stomach) in the form of partially purified powder.

Simulated tears: were purchased from Biochemazone and composed of Carboxymmethylcellulose, Sodium Bicarbonate, Potassium Chloride, Urea, Calcium Chloride, Sodium Chloride, Magnesium Chloride, Amino acids, Creatinine, Albumin, Glucose, Ascorbic acid, Citric Acid, Lactate, Glutathione, Vitamins, Agar, Hyaluronic acid, Pyruvate, Lysozyme, D-Glucose, G-Globulin, Bovine Serum Albumin, Calcium chloride dihydrate, Water Pyrogen Free water, Buffered solution, Additional Salts: $PO4^{3-}$, Na^{+}, K^{+}, Ca^{2+}, Cl^{-}, Viscosity Enhancers, Collagen. The pH value of the fluid was 7.4.

MUC 3 in simulated tears: In order to dissolve mucins in simulated tears (Biochemazone), the solution was centrifuged using a vortex (approximately 1 minute). The dilution of MUC 3 was 5 mg/mL. Then the sample was diluted to the next concentration of 0.2 mg/mL. For lasing experiments, the simulated tear sample and the sample with MUC 3 (0.2 mg/mL) dissolved in simulated tears were condensed according to the protocol described in Methods.

The samples were then subjected to lasing experiments described in methods.

For spectroscopic experiments (fluorescence and absorption), we used a more simplify model based on artificial tears (Thealoz Duo), which were composed of trehalose, sodium hyaluronate, sodium chloride, trometamol, hydrochloric acid, and water for injection. In order to dissolve mucins in artificial tears, the solution was centrifuged using a vortex (approximately 1 minute) and then the sample was filtered using 0.20 μm filters (CHROMAFIL Xtra OPTFE-20/25, 0.2 μm, 25 mm).

Methods:

Condensation process: For the condensation process we used 3kDa column filters (Amicon). First, we washed the filters (adding ~ 450 μl distilled water and centrifuging for about 10 minutes at 25 000 g). Then, we removed the water. In the next step, we added ~ 450 μl of the

sample (Biochemazone resembling tears with or without MUC 3) and centrifuged for 10 minutes at 25 000 g. We repeated this process 4 times, condensing a total of ~ 1500 μl of each sample (twice by adding 450 μl, twice 350 μl). Next, we performed an additional centrifugation. Finally, in order to obtain the condensed material, we turned the column and centrifuged for ~2.5 minutes at 1000 g.

UV-Vis Spectroscopy: Absorption spectra were measured using a CARY-5000 spectrophotometer with a 1 cm pathlength cuvette.

Steady-state and time-resolved fluorescence spectroscopy: For steady-state and time-resolved fluorescence measurements, femtosecond pulses were generated by frequency doubling in a BBO crystal, using the output of an optical parametric amplifier (Orpheus by Light Conversion) pumped by a femtosecond amplifier (Carbide by Light Conversion) at 420 nm. The pulse repetition rate was set at 2 MHz. Spectroscopic measurements were performed using a Horiba QuantaMaster 8075-11 spectrofluorometer, equipped with a PPD850 photomultiplier (sensitive from 250-850 nm) and a DeltaTime kit for time-resolved studies. Emission and excitation spectra were recorded from sample solutions in 1x1 cm quartz cuvettes. Both excitation and emission slits were set to 1 nm, and spectra were corrected for detector sensitivity. The power of the excitation beam was controlled to prevent saturation and ensure a linear detector response. The instrument response function (IRF) was determined by scattering the excitation beam in a TiO_2 suspension in water. Fluorescence decay data were analyzed using Horiba FelixGX software, applying a reconvolution method with the IRF for fitting multiexponential decay models to the experimental data. The average fluorescence lifetime was calculated as the amplitude-weighted mean of the decay times for each component.

Lasing in Fabry-Pérot Cavities: This term refers to a setup in which the liquid containing Thioflavin T (ThT) is sandwiched between two mirrors acting as photonic resonators. The mirror cavity provides strong optical feedback, enabling the detection of subtle molecular changes in the ThT-mucin complex. Lasing occurs when the excitation energy is gradually increased, achieved by incrementally adjusting the grey filter with a Thorlabs motor system, while simultaneously monitoring the emission spectrum with a detector. The relationship between pump energy and emission intensity was plotted to determine the lasing thresholds.

At a specific energy, the ThT emission spectrum narrows significantly, and a sharp increase in light intensity is observed. The pump energy at which this occurs is defined as the lasing threshold. Typically, when population inversion takes place within the cavity, a narrow lasing peak is observed, without background fluorescence. The full width at half-maximum (FWHM) of the lasing spectrum in the cavities is typically only a few nanometers wide.

Lasing spectra were recorded using a femtosecond laser operating at a 0.5 kHz repetition rate, with pulse energies of 400 μJ. The output beam was centered at 800 nm, and the wavelengths used for experiments were 420 nm.

The mirrors used for the cavities had nearly 100% transmission in the 400-450 nm range, while reflectance between 470-570 nm was approximately 95-99%, centered at 520-530 nm.

The lasing signal from the gain medium in the cavities was collected parallel to the excitation beam. A filter was used to block excitation wavelengths >470 nm, allowing only the lasing

signal to reach the detector. The lasing signal was collected using a ProEM Excelon camera from Teledyne Princeton Instruments.

We added 4 µl of sample and 2 µl of Tht at 25 mg/mL (dissolved in distilled water) to the mirror surface and then placed a cover mirror on top.

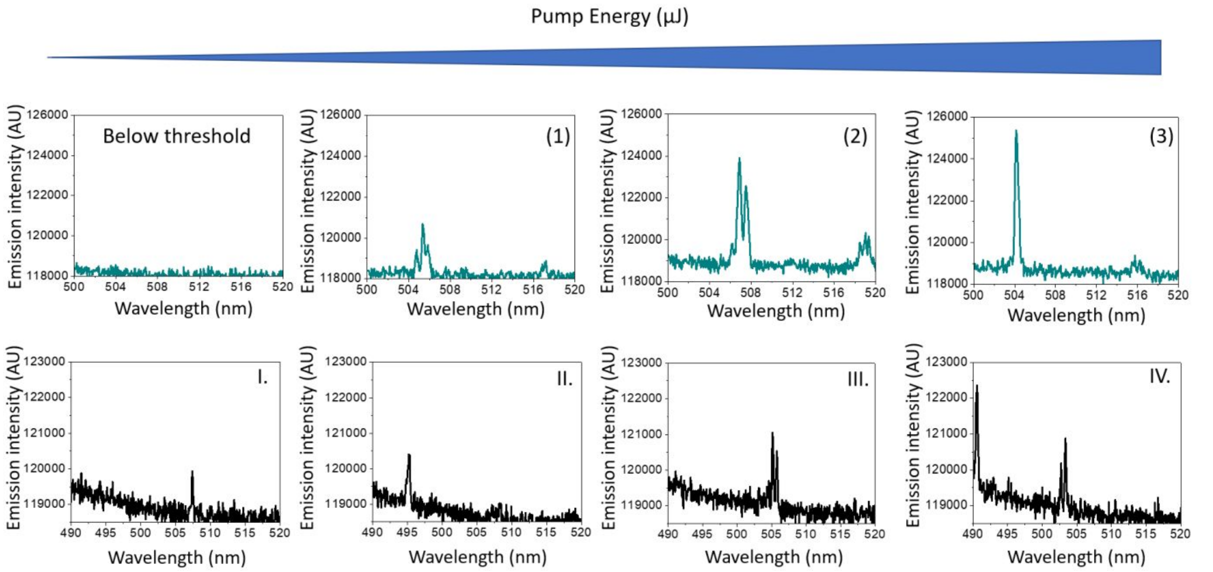

Fig. S1 *Lasing spectra of ThT in simulated tears as a function of pump energy. The top panel (cyan) displays the spectra for ThT - mucin complexes in simulated tears while the bottom panel (black) illustrates the spectra for ThT in simulated tears without mucins.*
